# Dual RNA-Seq analysis unveils the multifaceted mechanisms of *Trichoderma hamatum* in the biological control of *Fusarium graminearum*, the causal agent of wheat fusarium head blight

**DOI:** 10.3389/fmicb.2026.1742203

**Published:** 2026-01-16

**Authors:** Yunqing Cheng, Shuai Wang, Shuang Zhao, Siqi Yang, Yuqing Li, Bing Wang, Furan Zhang, Hongli He, Jianfeng Liu

**Affiliations:** Jilin Provincial Key Laboratory of Plant Resource Science and Green Production, Jilin Normal University, Siping, Jilin Province, China

**Keywords:** antagonistic interaction, differential gene expression, host-pathogen interaction, plant immunity priming, wheat disease management

## Abstract

**Background:**

Fusarium head blight (FHB), caused by *Fusarium graminearum* (Fg), is a devastating wheat disease leading to substantial yield losses. Effective biocontrol strategies are urgently needed.

**Objective:**

This study aimed to investigate the antagonistic potential of *Trichoderma hamatum* (Th) against Fg and elucidate its transcriptional mechanisms.

**Methods:**

Antagonistic activity was assessed via dual-culture and pot experiments with wheat seedlings under four treatments: control (CK), Th, Fg, and dual inoculation (Th-Fg). Transcriptome sequencing (RNA-seq) data were aligned to the genomes of Fg, Trichoderma guizhouense (as a proxy for Th), and *Triticum aestivum* to analyze gene expression changes.

**Results:**

Both assays showed Th strongly inhibited Fg growth. RNA-seq revealed that under Th pressure (Fg vs. Th–Fg), Fg exhibited widespread transcriptional suppression, with 608 DEGs downregulated and enriched in carbohydrate metabolism, indicating disrupted nutrient acquisition. Th itself showed minimal transcriptional changes (Th vs. Th–Fg), suggesting a resource-efficient strategy. Fg infection (CK vs. Fg) suppressed wheat photosynthesis and carbon metabolism, while Th inoculation (CK vs. Th) primed defense pathways. In the dual inoculation, Th alleviated Fg-induced suppression and enhanced wheat defense and physiological gene expression. qRT-PCR validated the RNA-seq reliability.

**Conclusion:**

*Trichoderma hamatum* suppresses Fg via a tripartite mechanism: direct antagonism by impairing pathogen metabolism, priming wheat immunity, and mitigating physiological damage. This study provides molecular insights for using *T. hamatum* as a potent biocontrol agent against FHB.

## 1 Introduction

Fusarium head blight (FHB), also known as wheat scab, is a devastating fungal disease that poses a major threat to global wheat production, leading to substantial yield losses and quality deterioration annually (Zhang et al., 2024; Nishiuchi and Kimura, 2021). Primarily caused by the ascomycete fungus *Fusarium graminearum*, FHB not only causes significant yield reductions but also contaminates grains with hazardous mycotoxins, such as deoxynivalenol (DON), which are detrimental to human and animal health (Fan et al., 2019). The management of FHB remains challenging due to the limited availability of highly resistant wheat cultivars and the variable efficacy, environmental concerns, and potential for fungicide resistance associated with chemical control (Moonjely et al., 2023). Therefore, the development of effective and sustainable alternative control strategies, particularly biological control, is urgently needed (Giedrojć et al., 2025).

Biological control using antagonistic microorganisms has emerged as a promising approach for integrated disease management (Dan-Dan et al., 2024). Among various biocontrol agents, fungi from the genus Trichoderma have been extensively studied and commercialized for their potent antagonistic abilities against a wide range of plant pathogens (Ren et al., 2025). Several Trichoderma species, such as *T. asperellum* and *T. harzianum*, have been reported to exhibit inhibitory effects against *F. graminearum* through multiple mechanisms, including mycoparasitism, antibiosis, and competition (Pierson et al., 2025; Mahmoud, 2016). However, despite their promising biocontrol potential, the field efficacy of *Trichoderma*strains can be inconsistent, influenced by environmental conditions and application strategies. For instance, their growth and biocontrol performance may be significantly limited in oligotrophic environments, such as wheat straw (Matarèse et al., 2012). Furthermore, the assessment of antagonistic potential can yield substantially different conclusions depending on the experimental approach (e.g., *in vitro* dual-culture vs. substrate colonization assays), highlighting the importance of systematic evaluation under more complex, plant-associated conditions (Schöneberg et al., 2015). While certain *Trichoderma*strains can induce systemic resistance in plants, such as ISR (Induced Systemic Resistance), thereby enhancing the host’s innate defense capabilities (Tiru et al., 2020), the specific mechanisms of *Trichoderma*spp. against FHB are often described in a fragmented manner. Studies typically focus either on the direct interaction between the antagonist and the pathogen (e.g., antagonism and mycotoxin inhibition) or separately on the plant’s induced responses. A comprehensive, simultaneous understanding of the tripartite interaction among the biocontrol agent, the pathogen, and the host plant at the molecular level is still largely lacking (Matarèse et al., 2012; Woo et al., 2023; Pedrero-Méndez et al., 2025; Khan et al., 2020). This gap hinders a holistic understanding of how *Trichoderma*functions within the complex plant-microbe interaction environment and limits the optimization of its application strategies. Within this context, *Trichoderma hamatum* (Th) has shown broad-spectrum biocontrol potential against various soil-borne diseases (Feng et al., 2025). Nevertheless, its efficacy, mode of action, and the underlying molecular mechanisms in the context of the air-borne pathogen *F. graminearum* causing FHB remain unexplored. Determining whether Th can suppress FHB through a multifaceted mechanism involving direct impairment of pathogen metabolism, activation of wheat defense responses, and alleviation of physiological damage constitutes a critical knowledge gap.

The objective of this study was to systematically evaluate the antagonistic potential of a *T. hamatum* strain against *F. graminearum* and to elucidate the underlying molecular mechanisms from the perspectives of all three interacting organisms. We hypothesized that *T. hamatum* suppresses *F. graminearum* and mitigates FHB through a multifaceted mechanism involving direct antagonism and indirect mediation of wheat defense responses. To test this hypothesis, we integrated *in vitro* dual-culture assays with in planta transcriptome sequencing (RNA-seq) analysis of a pot experiment involving four treatments: control (CK), *T. hamatum* inoculation (Th), *F. graminearum* inoculation (Fg), and dual inoculation (Th-Fg). By uniquely mapping the sequencing data to the genomes of *F. graminearum* (Basińska-Barczak et al., 2020; Cesarini et al., 2025), *Trichoderma guizhouense* (as a close proxy for *T. hamatum*; Korkom and Yıldız, 2023), and *Triticum aestivum* (Grujić et al., 2019), we aimed to simultaneously capture the global gene expression changes in the pathogen, the biocontrol agent, and the host plant. This integrated tripartite transcriptomic approach is expected to provide unprecedented insights into the complex interactions, revealing how *T. hamatum* directly impairs *F. graminearum*, how it primes the wheat immune system, and how it helps wheat alleviate physiological damage caused by the pathogen.

## 2 Materials and methods

### 2.1 Fungal strains and culture conditions

The fungal strains used in this study were *F. graminearum* (deposit No. CGMCC 3.3488) and *T. hamatum* (deposit No. CGMCC 20241). The *F. graminearum* strain was obtained from the China General Microbiological Culture Collection Center (CGMCC), while the *T. hamatum* strain was originally isolated, purified, and patented by our research group. The strains were initially activated on potato dextrose agar (PDA) plates. The PDA medium was prepared by boiling 200 g of peeled and diced potatoes in 1 L of distilled water for 30 min. The mixture was filtered through cheesecloth, and the filtrate was supplemented with 20 g of glucose (as the dextrose equivalent) and 15 g of agar, then adjusted to a final volume of 1 L with distilled water. The medium was sterilized by autoclaving at 121 °C for 20 min.

### 2.2 *In vitro* antagonism assay (dual-culture)

The antagonistic activity of *T. hamatum* (Th) against *F. graminearum* (Fg) was evaluated using a dual-culture assay on PDA medium, following a previously described method with modifications (Martínez-Padrón et al., 2023). Briefly, a mycelial plug (5 mm in diameter) taken from the actively growing margin of a 3-day-old *F. graminearum* culture was placed at the center of a fresh PDA plate. Four mycelial plugs of the same size from a 5-day-old *T. hamatum* culture were then inoculated symmetrically at the four corners of the plate, each approximately 3 cm from the central plug. Control plates were inoculated with only a *F. graminearum* plug at the center. All plates were sealed with Parafilm and incubated at 25 °C in the dark. The experiment included three independent biological replicates, each consisting of three technical replicate plates (*n* = 9). The radial growth of *F. graminearum* toward the *T. hamatum* colonies was monitored daily. After 5–7 days of incubation, the antagonistic effect was assessed by measuring the inhibition of radial growth in the dual-culture plates compared to the control plates. The percentage inhibition of mycelial growth (PIMG) was calculated as follows: PIMG (%) = [(Rc – Rt) / Rc] × 100, where Rc represents the radial growth of the pathogen in the control plate, and Rt represents the radial growth in the dual-culture plate (Yassin et al., 2021). The interaction zone between the two fungi was also visually examined for signs of mycoparasitism, such as hyphal coiling and lysis.

### 2.3 In planta antagonism assay and sample collection for transcriptome analysis

Plant growth and experimental design. Wheat seeds (*Triticum aestivum* L. cv. Longmai 35) were sown in plastic pots containing a sterile humus substrate, with 20 seeds per pot (12 pots total). Plants were grown in a controlled climate chamber at 28 °C under a 16/8 h light/dark cycle and 70% relative humidity for 23 days until the seedling stage. The experiment included four treatments: (1) mock-inoculated control (CK, wounded but not inoculated); (2) *F. graminearum* inoculation (Fg); (3) *T. hamatum* inoculation (Th); and (4) co-inoculation of *F. graminearum* and *T. hamatum* (Th–Fg). Each treatment consisted of three biological replicates, with each replicate comprising three individual seedling stems (nine stems per treatment in total).

Preparation of fungal inocula. *F. graminearum* and *T. hamatum* were pre-cultured on PDA medium for 7 days. Mycelial plugs (0.2 cm × 0.2 cm) free of agar were collected from the actively growing margins using a sterile scalpel. Approximately 20 plugs of each fungus were transferred into 300 mL of potato dextrose broth (PDB) and incubated at 28 °C with shaking at 180 rpm for 72 h to form compact mycelial pellets (approximately 0.6 cm in diameter). The pellets were harvested by centrifugation at 5,000 × g for 10 min and washed twice with sterile saline under the same conditions. One mycelial pellet was used to inoculate each wheat seedling.

Seedling inoculation. A longitudinal wound (approximately 1 cm long and less than 50% of the stem diameter in depth) was made on the lowest internode of each seedling using a sterile scalpel. Depending on the treatment, the wound was inoculated with one pellet of Fg, one pellet of Th, or one pellet each of Fg and Th. Control seedlings were wounded but not inoculated.

Disease assessment and sample collection. After inoculation, the treated stem segments were placed on moist filter paper in Petri dishes to maintain humidity and promote disease development. Disease symptoms were monitored daily. Five days post-inoculation, when lesions were visible in Fg-treated plants, a 2-cm stem segment surrounding the inoculation site was excised from each seedling. For each biological replicate, segments from three seedlings were pooled, flash-frozen in liquid nitrogen, and stored at −80 °C for RNA extraction. The entire experiment was repeated independently to ensure reproducibility.

### 2.4 RNA extraction, library construction, and transcriptome sequencing

RNA extraction and quality control. Total RNA was extracted from approximately 100 mg of frozen wheat stem segments using the RNAprep Pure Plant Kit (Tiangen, China) according to the manufacturer’s instructions. RNA concentration and purity were measured using a NanoDrop spectrophotometer and a Qubit 2.0 Fluorometer, respectively. RNA integrity was assessed using an Agilent 2,100 Bioanalyzer. Only RNA samples with an OD260/280 ratio of 1.8–2.2, an OD260/230 ratio > 2.0, and an RNA integrity number (RIN) > 7.0 were used for library construction.

Library construction and sequencing. RNA-seq library preparation and sequencing were performed by Beijing Biomarker Technologies Co., Ltd. Briefly, mRNA was isolated from total RNA using oligo(dT)-attached magnetic beads and randomly fragmented. First-strand cDNA was synthesized using random hexamer primers, followed by second-strand synthesis. The double-stranded cDNA was purified with AMPure XP beads, end-repaired, A-tailed, and ligated to adapters. Fragments of 300–400 bp were selected and PCR-amplified to construct the final libraries. Library quality was assessed using an Agilent 2,100 Bioanalyzer and quantified by Qubit 2.0 and qPCR. Libraries with concentrations above 2 nM were sequenced on an Illumina platform NovaSeq 6,000 in paired-end 150 bp (PE150) mode.

### 2.5 Bioinformatic analysis of RNA-Seq data

Data processing and alignment. Raw paired-end reads were processed with fastp (v0.20.0; Chen et al., 2018) to remove adapters, poly-N sequences, and low-quality bases, yielding high-quality clean data. Clean reads were aligned separately to the following reference genomes using HISAT2 (v2.2.1; Kim et al., 2015): *Triticum aestivum* (IWGSC RefSeq v2.1; assembly: Triticum_aestivum.v2.1.genome.fa; Plavšin et al., 2021), *F. graminearum* (strain PH-1; assembly: Fusarium_graminearum_PH-1. ASM24013v3.genome.fa; King et al., 2017), and *Trichoderma guizhouense* (assembly: Trichoderma_guizhouense. ASM202278v1.genome.fa; Zhu et al., 2021), used as a proxy for *T. hamatum* due to high genomic similarity.

Transcriptome assembly and quantification. Mapped reads were assembled into transcripts, and their abundances were estimated using StringTie (v2.2.1; Pertea et al., 2015; Shumate et al., 2022) in a reference-based manner. FPKM (Fragments Per Kilobase of transcript per Million fragments mapped) values were calculated by StringTie for transcript-level quantification.

In the equation, cDNA Fragments represents the number of PE reads mapped to the specific transcript; Mapped Fragments (Millions) is the number of all mapped reads, which is counted as 10^6^; Transcript Length(kb) is the length of transcript in unit of 10^3^b. Differential expression analysis. Read counts per gene were used for differential expression analysis with DESeq2 (v1.30.1; Liu et al., 2021). Genes with an adjusted *p*-value (FDR) < 0.05 and |log₂(fold change)| > 1 were considered differentially expressed (DEGs). Functional enrichment analysis. Gene Ontology (GO) and Kyoto Encyclopedia of Genes and Genomes (KEGG) pathway enrichment analyses of DEGs were performed using clusterProfiler (v4.0.5; Xu et al., 2024; Yu, 2024). Terms with a corrected *p*-value < 0.05 were deemed significantly enriched.

### 2.6 Quantitative real-time PCR (qRT-PCR) validation

To validate the RNA-seq results, qRT-PCR was performed on 30 differentially expressed genes (DEGs)—10 from *T. hamatum*, 10 from *F. graminearum*, and 10 from *T. aestivum*—selected from the most significantly enriched KEGG pathways in the respective comparisons (e.g., Fg vs. Th–Fg for *F. graminearum*; Th vs. Th–Fg for *T. hamatum*; CK vs. Fg/Th/Th–Fg for wheat). The same RNA samples used for RNA-seq were reverse-transcribed into cDNA using the PrimeScript RT reagent Kit with gDNA Eraser (TaKaRa, Japan). qRT-PCR was conducted on a QuantStudio 5 system (Applied Biosystems, United States) with TB Green Premix Ex Taq II (TaKaRa, Japan). Each 20 μL reaction contained 10 μL of TB Green Premix, 0.8 μL each of forward and reverse primers (10 μM), 2 μL of cDNA, and 6.4 μL of nuclease-free water. The thermal profile was: 95 °C for 30 s; 40 cycles of 95 °C for 5 s and 60 °C for 30 s. Melt curve analysis confirmed amplification specificity. All reactions were run in three technical replicates per biological replicate. Primer sequences are listed in Supplementary Table S1. The reference genes used for normalization were *actin* for *T. aestivum*, *β-tubulin* for *F. graminearum*, and *tef1* for *T. hamatum*. Relative expression was calculated using the 2 ^–ΔΔCt^ method (Schmittgen and Livak, 2008).

## 3 Results

### 3.1 *Trichoderma Hamatum* exhibits strong *in vitro* antagonistic activity against *Fusarium graminearum*

**Figure 1:**
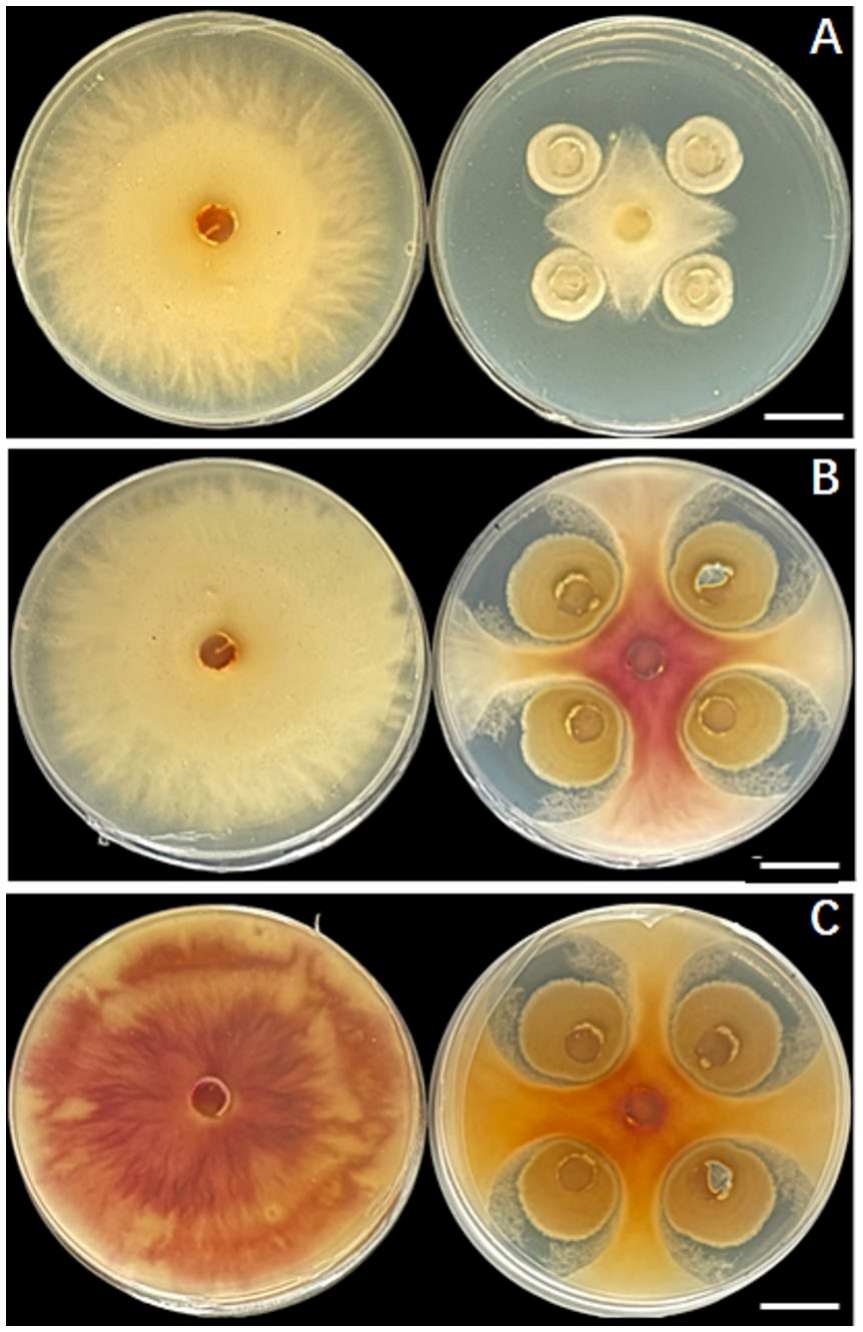
*In vitro* antagonistic activity of *Trichoderma hamatum* against *Fusarium graminearum* in a dual-culture assay. **(A–C)** Representative images of the confrontation assay at 3, 5, and 7 days post-inoculation (dpi), respectively. For each panel, the left plate shows the control inoculated with a single plug of *F. graminearum* (Fg) at the center, while the right plate shows the dual-culture treatment inoculated with Fg at the center and four plugs of *T. hamatum* (Th) equidistantly at the corners. Scale bars: 2.0 cm.

The direct antagonistic capability of *T. hamatum* against *F. graminearum* was first evaluated using a dual-culture assay. As depicted in Figure 1, a clear and time-dependent inhibitory effect was observed. In the control plates, *F. graminearum* grew radially, forming uniform colonies at 3-, 5-, and 7-days post-inoculation (dpi; Figures 1A–C). In stark contrast, the co-culture with *T. hamatum* resulted in a progressive suppression of the pathogen’s growth. By 5 dpi, a distinct inhibition zone became apparent (Figure 1B), which further expanded by 7 dpi. Quantitative analysis revealed that *T. hamatum* inhibited the mycelial growth of *F. graminearum* with an efficacy of 51.1% based on the percentage inhibition of mycelial growth (PIMG), calculated from the radial growth measurements. Notably, at 7 dpi, the interaction zone exhibited a prominent color change to red (Figure 1C), suggesting a severe disruption of the pathogen’s metabolic activity. This result is consistent with previous studies, which have reported significant *in vitro* antagonism of various *Trichoderma* species against *F. graminearum* (Pierson et al., 2025; Mahmoud, 2016). These visual and quantitative results unequivocally demonstrate the potent in vitro antagonistic activity of *T. hamatum*against *F. graminearum*, providing a phenotypic foundation for the subsequent molecular investigations.

### 3.2 *Trichoderma Hamatum* alleviates *Fusarium graminearum*-induced symptoms in wheat seedlings

**Figure 2:**
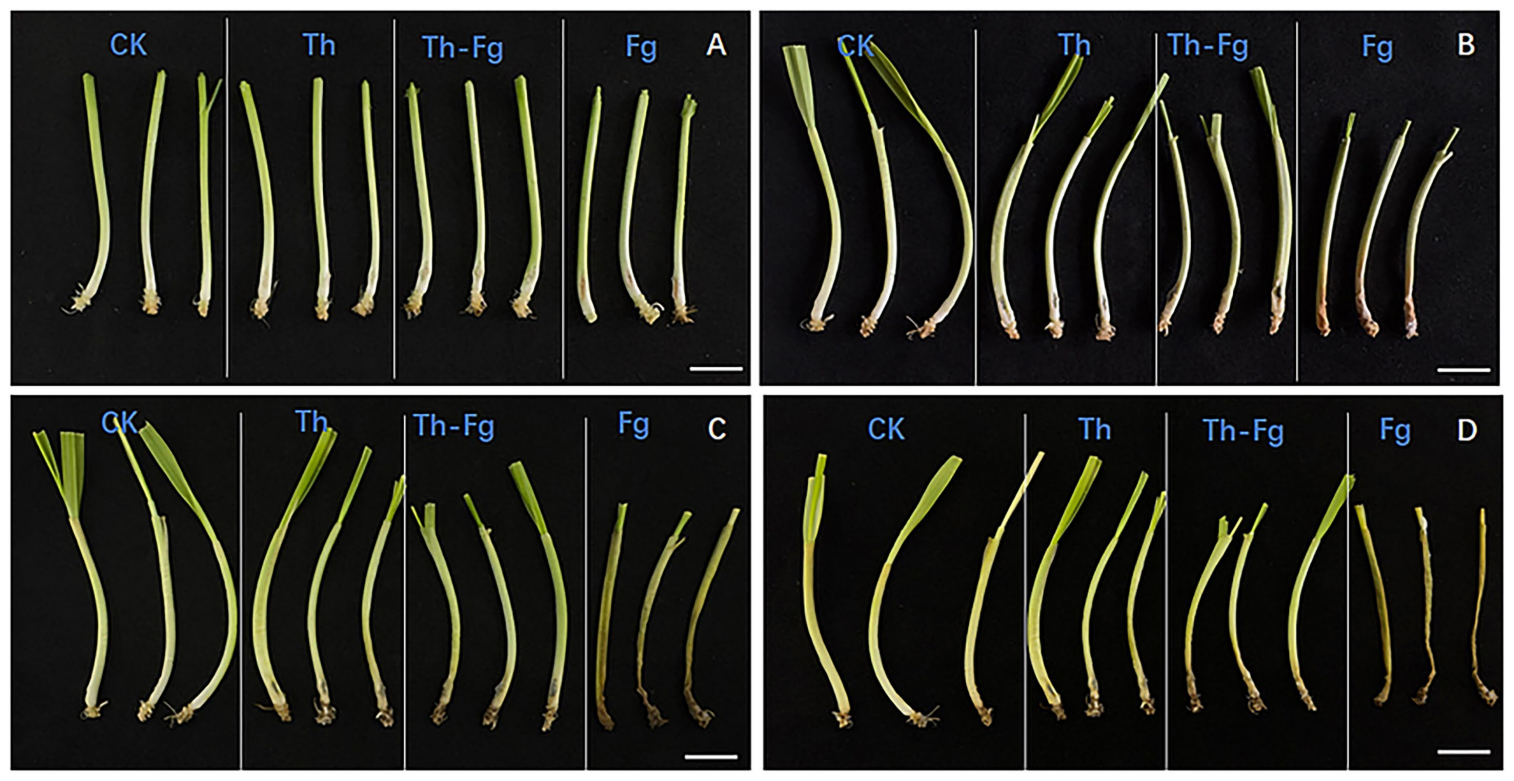
*In planta* antagonistic activity of *Trichoderma hamatum* against *Fusarium graminearum* on wheat seedlings. **(A–D)** Disease symptoms on wheat seedling stems at 1, 3, 5, and 7 days post-inoculation (dpi), respectively. The treatments are as follows: CK, mock-inoculated control (wounded but not inoculated); Fg, inoculated with *F. graminearum* alone; Th, inoculated with *T. hamatum* alone; Th-Fg, co-inoculated with both *F. graminearum* and *T. hamatum*. Scale bars: 2.0 cm.

The *in planta* biocontrol efficacy of *T. hamatum* was evaluated on wheat seedlings (Figure 2). Over a 7-day period, distinct disease progression was observed across the treatments. Seedlings inoculated with *F. graminearum* (Fg) alone developed severe necrotic lesions at the inoculation site, which rapidly expanded along the stem. In contrast, seedlings co-inoculated with *T. hamatum* (Th-Fg) exhibited markedly reduced disease symptoms. The lesions in the Th-Fg treatment were significantly smaller and less severe compared to the Fg-alone treatment at each time point, demonstrating the potent protective effect of *T. hamatum*. Notably, seedlings mock-inoculated (CK) or inoculated with *T. hamatum* alone (Th) showed no visible symptoms, confirming that the pathogenicity was solely attributable to *F. graminearum* and that *T. hamatum* itself was non-pathogenic to wheat. These results clearly indicate that *T. hamatum* effectively suppresses disease development caused by *F. graminearum in vivo*.

### 3.3 Sequencing data quality and efficient mapping to the tripartite genomes

To elucidate the transcriptional dynamics underlying the tripartite interaction, we performed RNA-seq on stem samples from all four treatments (CK, Fg, Th, Th-Fg), with three biological replicates per treatment. A total of 12 libraries were constructed and sequenced, generating an average of 54.6 million high-quality clean reads per sample, with Q30 scores exceeding 94.95% and GC contents ranging from 52.47 to 54.52%, indicating high sequencing quality (Supplementary Table S2). A tripartite mapping strategy was employed, wherein the clean reads were separately and simultaneously aligned to the reference genomes of *Triticum aestivum*(host), *F. graminearum* (pathogen), and *Trichoderma guizhouense* (as a proxy for the biocontrol agent, *T. hamatum*). This approach was chosen to directly and independently capture the concurrent transcriptional responses of all three interacting organisms from the mixed RNA samples, which is essential for dissecting the complex molecular crosstalk within the tripartite system. The clean reads were then separately aligned to the reference genomes of *Triticum aestivum*, *F. graminearum*, and *Trichoderma guizhouense* (as a proxy for *T. hamatum*). As expected, the vast majority of reads (80.04 to 93.83%) mapped uniquely to the wheat genome across all samples, confirming the plant origin of most transcripts (Supplementary Table S5). Notably, a substantial proportion of reads from the Fg and Th-Fg samples mapped to the *F. graminearum* genome, confirming successful pathogen colonization (Supplementary Table S3). In contrast, the mapping rate to the *T. guizhouense* genome was exceptionally low (≤ 0.05%) in all samples, including the Th and Th-Fg treatments (Supplementary Table S4). This suggests that the biomass of *T. hamatum* in the stem tissues were minimal at the sampling time point, a finding consistent with its role as a rhizosphere-colonizing biocontrol agent rather than an endophyte. The high-quality sequencing data and species-specific mapping efficiency provided a robust foundation for the subsequent differential gene expression analysis.

### 3.4 *Trichoderma Hamatum* triggers extensive transcriptional suppression in *Fusarium graminearum*, disrupting key metabolic pathways

**Figure 3:**
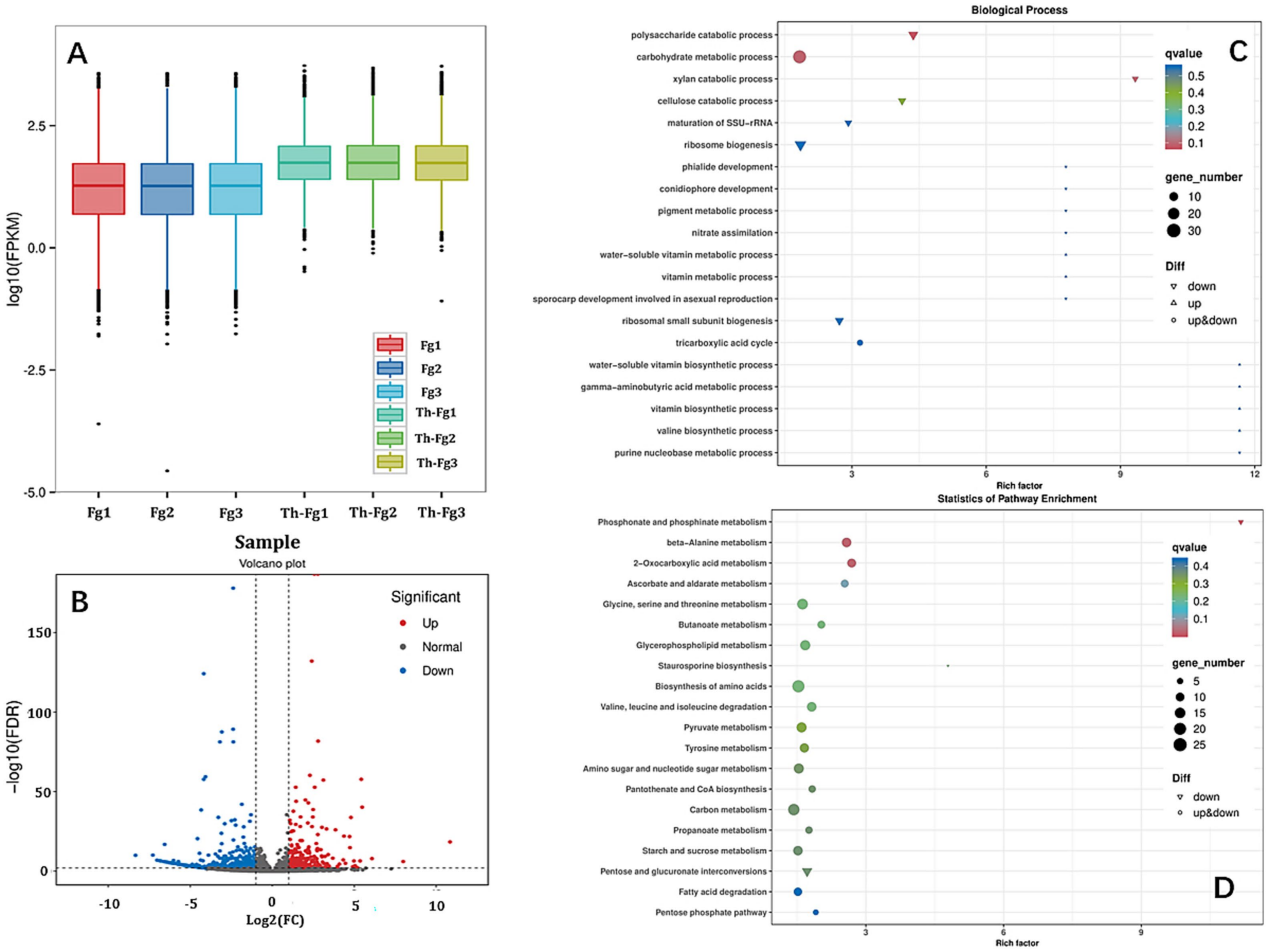
Transcriptomic profiling of *Fusarium graminearum* under the antagonism of *Trichoderma hamatum* reveals significant suppression of metabolic pathways. **(A)** FPKM boxplot of each sample. X-axis: Sample IDs; Y-axis: log_10_(FPKM); this plot shows the overall expression level of each sample via the dispersion of gene expression in each sample. **(B)** Volcano plot of differentially expressed genes (DEGs) identified in the *F. graminearum* (Fg) vs. dual inoculation (Th-Fg) comparison. Each dot represents a gene. The X-axis shows the log_2_(Fold Change), and the Y-axis shows the -log_10_(False Discovery Rate, FDR). Dots farther from the Y-axis (X = 0) indicate a greater magnitude of expression change; dots farther from the X-axis (Y = 0) indicate higher statistical significance. **(C)** Gene ontology (GO) enrichment analysis (biological process) of the DEGs. **(D)** Kyoto encyclopedia of genes and genomes (KEGG) pathway enrichment analysis of the DEGs. The experimental treatments were defined as follows: CK, mock-inoculated control; Fg, inoculated with *F. graminearum* alone; Th, inoculated with *T. hamatum* alone; Th-Fg, co-inoculated with both fungi. All RNA-seq reads were uniquely mapped to the *F. graminearum* reference genome for this analysis.

To dissect the molecular basis of the observed antagonism, we analyzed the transcriptomic response of *F. graminearum* when challenged by *T. hamatum* (Fg vs. Th-Fg). The overall gene expression profile, as shown by the FPKM density distribution, indicated a shift in the transcriptome of *F. graminearum* in the presence of the antagonist (Figure 3A). Comparative analysis identified a total of 889 differentially expressed genes (DEGs; for a complete list, see Supplementary Table S6), with a strong bias toward downregulation (608 genes downregulated vs. 281 upregulated), demonstrating that *T. hamatum* predominantly suppresses the transcriptional activity of the pathogen (Figure 3B). Functional enrichment analysis of these DEGs provided critical insights into the processes being targeted. GO analysis (Biological Process) revealed that the downregulated genes were significantly enriched in terms related to carbohydrate metabolism, including “polysaccharide catabolic process,” “xylan catabolic process,” and “carbohydrate metabolic process” (Figure 3C). This suggests that *T. hamatum* impairs the pathogen’s ability to acquire nutrients and maintain cell wall integrity. Furthermore, KEGG pathway analysis showed significant enrichment of DEGs in metabolic pathways such as “Phosphonate and phosphinate metabolism,” “beta-Alanine metabolism,” and “2-Oxocarboxylic acid metabolism” (Figure 3D). Collectively, these transcriptomic findings indicate that the direct antagonistic effect of *T. hamatum* is mediated, at least in part, by a widespread suppression of key metabolic processes essential for *F. graminearum* growth and virulence.

### 3.5 *Trichoderma Hamatum* exhibits minimal transcriptional reprogramming when confronting *Fusarium graminearum*, suggesting a resource-efficient antagonistic strategy

**Figure 4:**
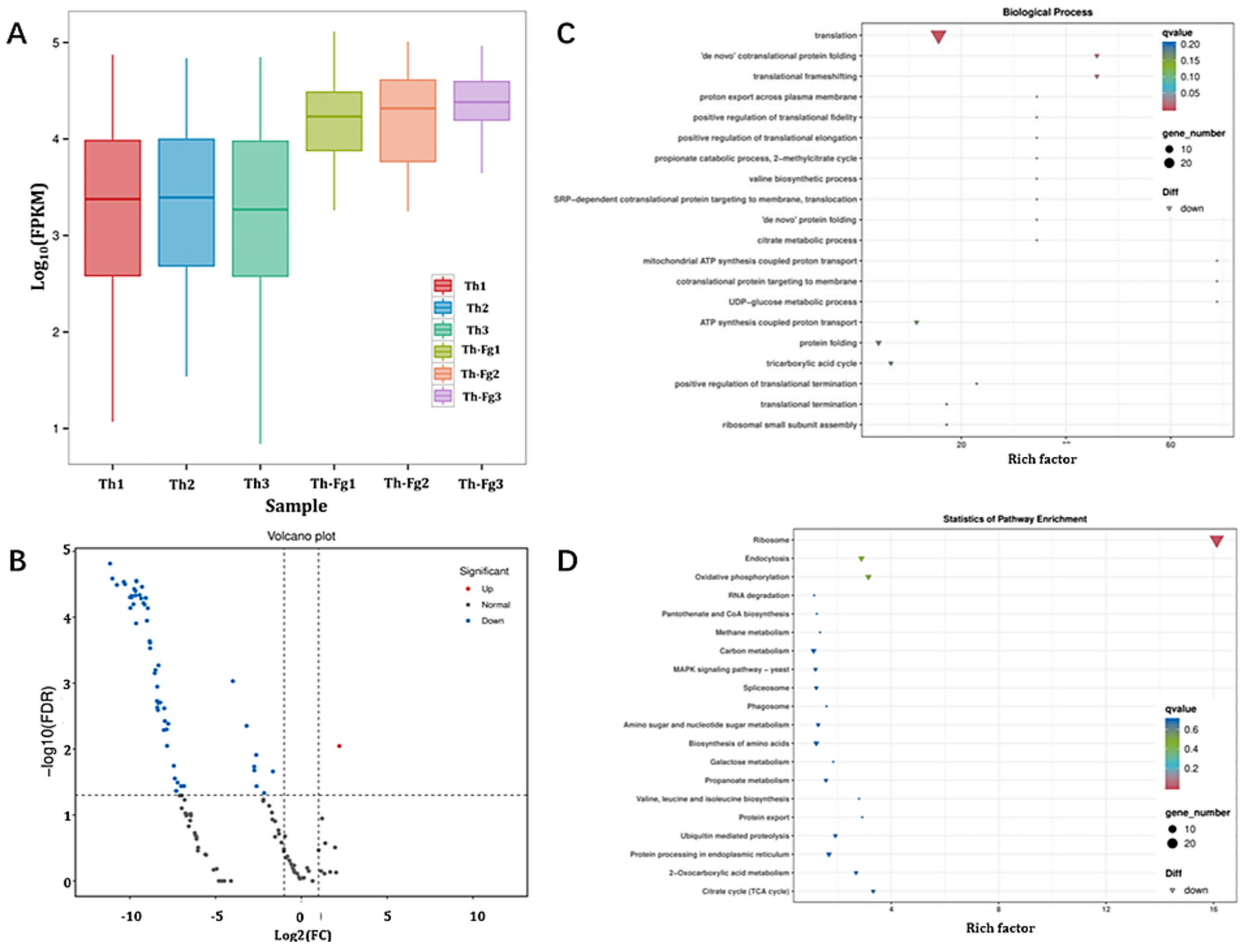
Transcriptomic response of *Trichoderma hamatum* to co-culture with *Fusarium graminearum* reveals minimal transcriptional reprogramming. **(A)** FPKM boxplot of each sample. X-axis: Sample IDs; Y-axis: log_10_(FPKM); this plot shows the overall expression level of each sample via the dispersion of gene expression in each sample. **(B)** Volcano plot of differentially expressed genes (DEGs) identified in the *T. hamatum* (*Th*) vs. dual inoculation (*Th-Fg*) comparison. The X-axis shows the log_2_(Fold Change), and the Y-axis shows the -log_10_(False Discovery Rate, FDR). **(C)** Gene ontology (GO) enrichment analysis (biological process) of the DEGs. **(D)** Kyoto encyclopedia of genes and genomes (KEGG) pathway enrichment analysis of the DEGs. The experimental treatments were defined as follows: CK, mock-inoculated control; *Th*, inoculated with *T. hamatum* alone; *Fg*, inoculated with *F. graminearum* alone; *Th-Fg*, co-inoculated with both fungi. All RNA-seq reads were uniquely mapped to the *Trichoderma guizhouense* reference genome (as a proxy for *T. hamatum*) for this analysis.

In stark contrast to the extensive transcriptional alterations observed in *F. graminearum*, the transcriptomic profile of *T. hamatum* itself remained largely stable upon confrontation with the pathogen. The FPKM density distribution showed highly overlapping curves between the Th and Th-Fg treatments, indicating minimal global changes in gene expression (Figure 4A). Comparative analysis (Th vs. Th-Fg) identified only 61 differentially expressed genes (DEGs; see Supplementary Table S7), a remarkably small number, with 60 genes being downregulated and a single gene upregulated (Figure 4B). Functional enrichment analysis of this limited set of DEGs revealed that the most significantly affected biological processes were all related to translation, including “translation,” “translational frameshifting,” and “‘*de novo*’ cotranslational protein folding” (Figure 4C). Accordingly, KEGG pathway analysis confirmed a significant enrichment in the “Ribosome” pathway (ko03010; Figure 4D). This highly focused transcriptional response, centered on the protein synthesis machinery, suggests that *T. hamatum* does not undergo a massive metabolic reorganization to combat *F. graminearum*. Instead, it may rely on pre-formed or constitutively expressed antagonistic machinery, representing a resource-efficient biocontrol strategy that allows for a rapid and energetically favorable response to the pathogen.

### 3.6 *Fusarium graminearum* infection causes extensive transcriptional suppression in wheat, disrupting primary metabolism and defense responses

**Figure 5:**
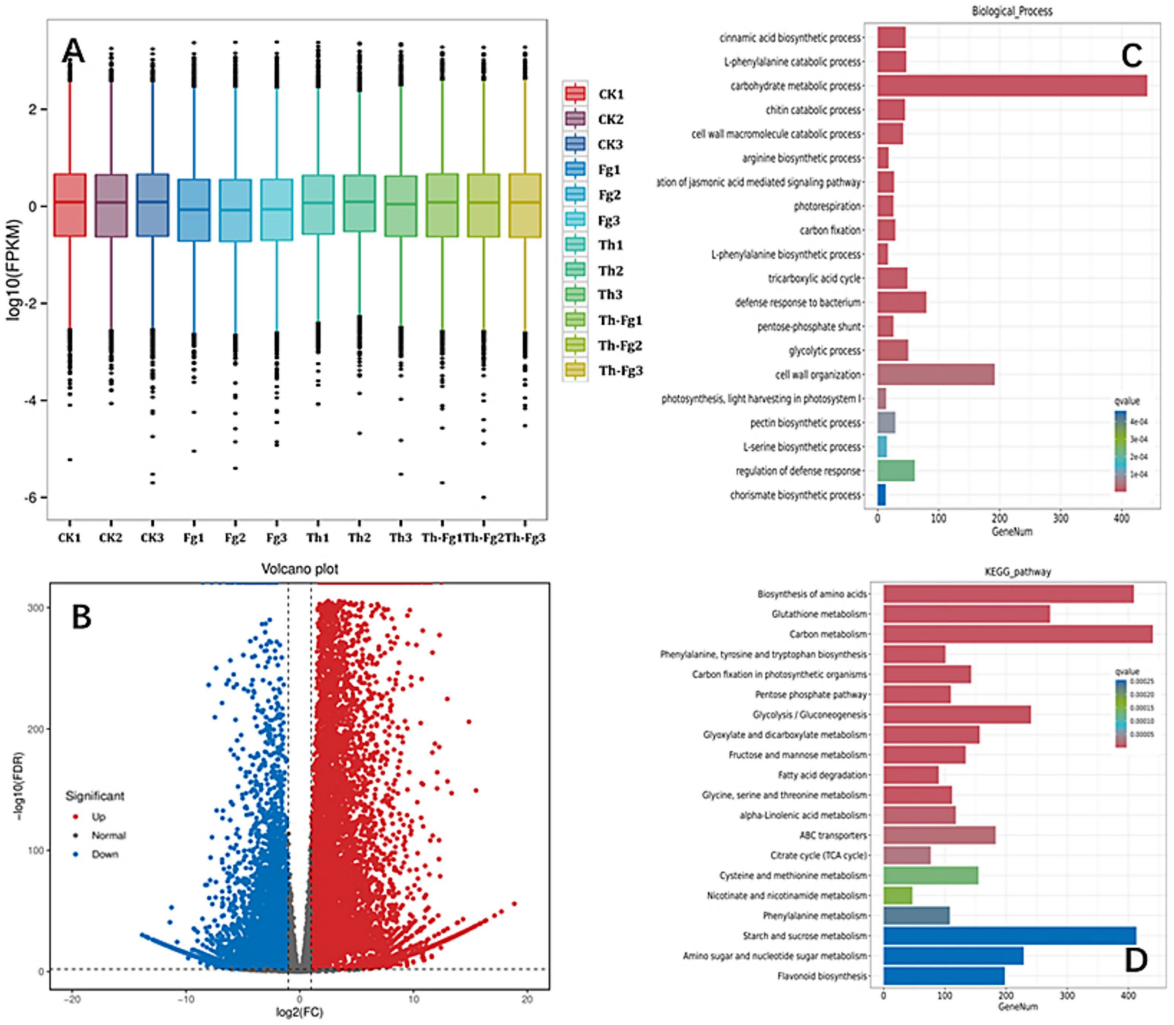
Transcriptomic profiling of wheat (*Triticum aestivum*) reveals physiological suppression by *Fusarium graminearum*. **(A)** FPKM boxplot of each sample. X-axis: sample IDs; Y-axis: log10(FPKM); this plot shows the overall expression level of each sample via the dispersion of gene expression in each sample. **(B)** Volcano plot of differentially expressed genes (DEGs) identified in the comparison between mock-inoculated control (CK) and *F. graminearum* inoculation (Fg). **(C)** Gene ontology (GO) enrichment analysis (biological process) of the DEGs from the CK vs. Fg comparison. **(D)** Kyoto encyclopedia of genes and genomes (KEGG) pathway enrichment analysis of the DEGs. The experimental treatments were defined as follows: CK, mock-inoculated control; Th, inoculated with *T. hamatum* alone; Fg, inoculated with *F. graminearum* alone; Th-Fg, co-inoculated with both fungi. All RNA-seq reads were uniquely mapped to the *Triticum aestivum* reference genome for this analysis.

We next investigated the impact of *F. graminearum* infection on the wheat transcriptome. The global gene expression profile revealed a pronounced leftward shift of the FPKM density curve for the Fg treatment compared to the control (CK), indicating a widespread suppression of gene expression in pathogen-inoculated plants (Figure 5A). This massive transcriptional alteration was quantified in the CK vs. Fg comparison, which identified 23,526 differentially expressed genes (DEGs; for a complete list, see Supplementary Table S8), underscoring the profound disruptive effect of the pathogen (Figure 5B). GO enrichment analysis showed that these DEGs were significantly involved in critical biological processes, including the biosynthesis of defense-related compounds like cinnamic acid, carbohydrate metabolism, and cell wall macromolecule catabolism (Figure 5C). KEGG pathway analysis further demonstrated that the infection significantly affected pathways central to plant physiology, such as “Biosynthesis of amino acids,” “Carbon metabolism,” “Carbon fixation in photosynthetic organisms,” and “Glutathione metabolism” (Figure 5D). These findings indicate that *F. graminearum* infection imposes severe physiological stress on wheat, repressing fundamental metabolic activities including photosynthesis and energy production, while simultaneously triggering complex but potentially dysregulated defense responses.

### 3.7 *Trichoderma hamatum* primes the wheat immune system by activating defense-related pathways in the absence of the pathogen

**Figure 6:**
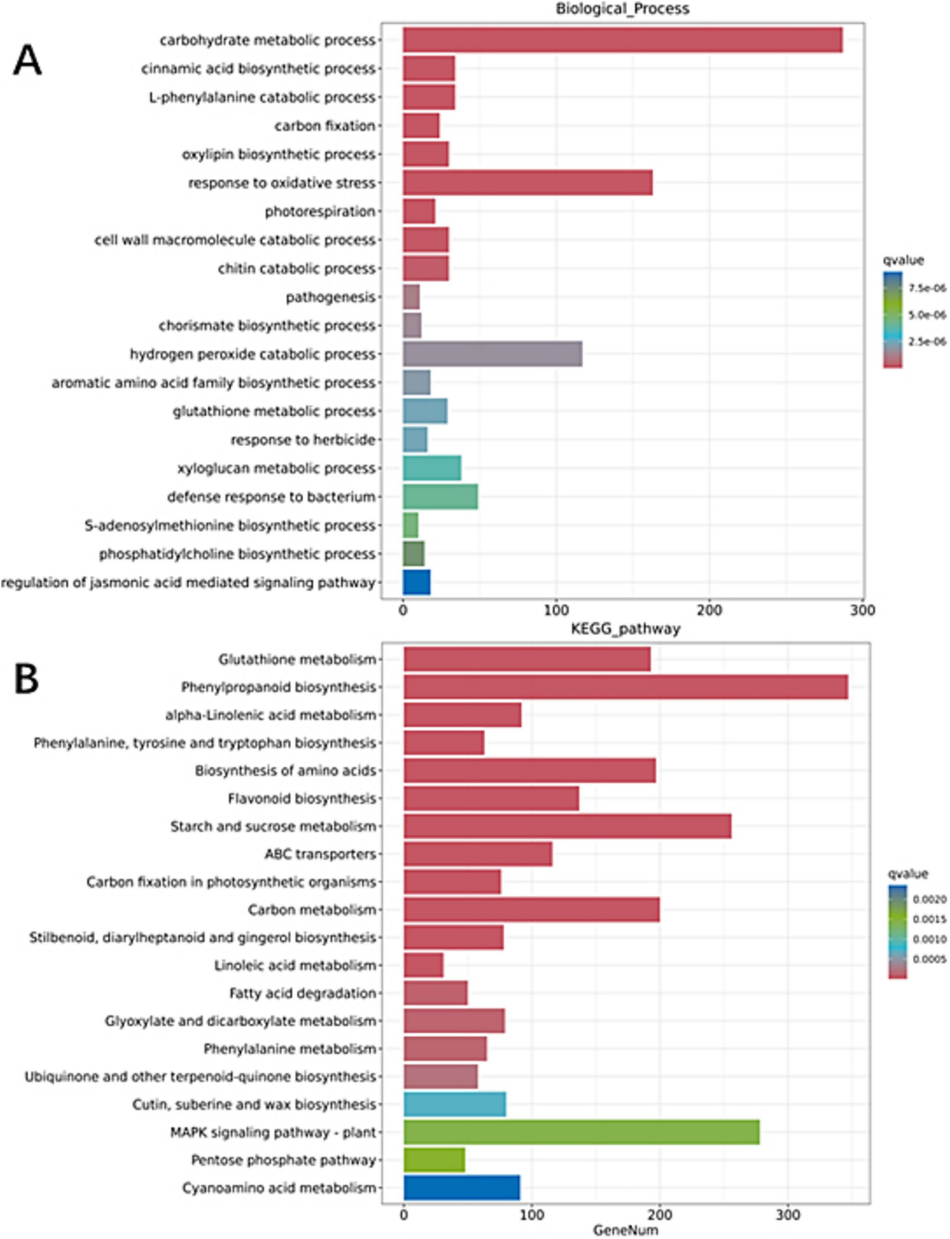
Transcriptomic analysis reveals the priming effect of *Trichoderma hamatum* on wheat defense responses. **(A)** Gene ontology (GO) enrichment analysis (biological process) of the differentially expressed genes (DEGs) identified in the comparison between mock-inoculated control (CK) and *T. hamatum* inoculation (Th). **(B)** Kyoto encyclopedia of genes and genomes (KEGG) pathway enrichment analysis of the DEGs from the CK vs. Th comparison. The experimental treatments were defined as follows: CK, mock-inoculated control; Th, inoculated with *T. hamatum* alone; Fg, inoculated with *F. graminearum* alone; Th-Fg, co-inoculated with both fungi. All RNA-seq reads were uniquely mapped to the *Triticum aestivum* reference genome for this analysis.

To determine whether *T. hamatum* alone could induce a defense response in wheat, we analyzed the transcriptome of plants inoculated only with the biocontrol agent (CK vs. Th). This comparison identified 11,711 differentially expressed genes (DEGs; see Supplementary Table S9), indicating that *T. hamatum* inoculation significantly reprograms the wheat transcriptome even in the absence of *F. graminearum*. Functional enrichment analysis of these DEGs revealed a distinct pattern compared to the pathogen response. GO terms significantly enriched were related to “carbohydrate metabolic process,” “cinnamic acid biosynthetic process,” and “L-phenylalanine catabolic process” (Figure 6A). More importantly, KEGG pathway analysis showed a strong activation of well-characterized defense signaling and biosynthesis pathways, most notably “Phenylpropanoid biosynthesis,” “Glutathione metabolism,” and “alpha-Linolenic acid metabolism” (Figure 6B). The upregulation of phenylpropanoid biosynthesis is associated with the production of antimicrobial compounds like flavonoids and lignin, while glutathione and alpha-linolenic acid metabolism are integral to redox homeostasis and jasmonic acid signaling, respectively. These results demonstrate that *T. hamatum* acts as a potent priming agent, pre-activating a broad spectrum of the wheat defense arsenal and preparing the plant for a more efficient response to subsequent pathogen challenge.

### 3.8 Co-inoculation with *Trichoderma Hamatum* alleviates *Fusarium graminearum*-induced physiological suppression and enhances defense responses in wheat

**Figure 7:**
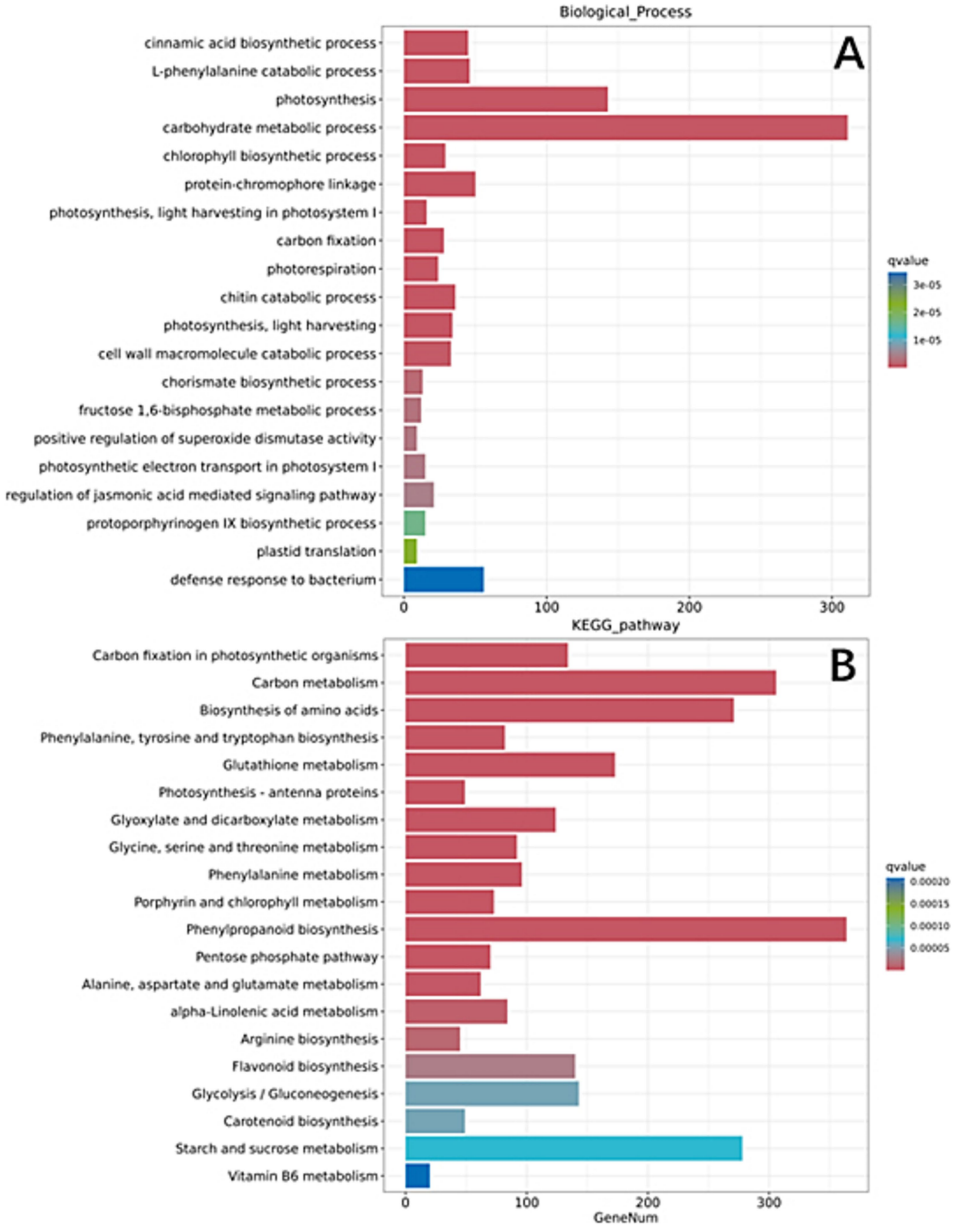
Transcriptomic analysis reveals the synergistic effect of *Trichoderma hamatum* in alleviating *Fusarium graminearum*-induced suppression and restoring wheat physiological processes. **(A)** Gene Ontology (GO) enrichment analysis (biological process) of the differentially expressed genes (DEGs) identified in the comparison between mock-inoculated control (CK) and dual inoculation (Th-Fg). **(B)** Kyoto Encyclopedia of Genes and Genomes (KEGG) pathway enrichment analysis of the DEGs from the CK vs. Th-Fg comparison. The experimental treatments were defined as follows: CK, mock-inoculated control; Th, inoculated with *T. hamatum* alone; Fg, inoculated with *F. graminearum* alone; Th-Fg, co-inoculated with both fungi. All RNA-seq reads were uniquely mapped to the *Triticum aestivum* reference genome for this analysis.

The transcriptomic profile of wheat under dual inoculation (Th-Fg) revealed the integrated outcome of the tripartite interaction. Comparison with the control (CK vs. Th-Fg) identified 14,958 differentially expressed genes (DEGs; for a complete list, see Supplementary Table S10). Strikingly, the functional characteristics of these DEGs differed markedly from those induced by *F. graminearum* alone. GO enrichment analysis highlighted the significant upregulation of processes including “cinnamic acid biosynthetic process,” “L-phenylalanine catabolic process,” and, most notably, “photosynthesis” (Figure 7A). This indicates that the presence of *T. hamatum* not only maintained the activation of defense-related pathways but also counteracted the pathogen’s suppression of core physiological functions. KEGG pathway analysis further supported this conclusion, showing significant enrichment in “Carbon fixation in photosynthetic organisms,” “Carbon metabolism,” and “Biosynthesis of amino acids” (Figure 7B). The enhanced expression of genes in these pathways suggests that *T. hamatum* effectively mitigates the severe physiological damage caused by *F. graminearum*, helping to restore the plant’s energy production and primary metabolism. Concurrently, the sustained enrichment of phenylpropanoid-related terms points to a robust, ongoing defense activation. These results demonstrate that *T. hamatum* orchestrates a protective transcriptional reprogramming in wheat that simultaneously reinforces defense capabilities and promotes physiological recovery from pathogen stress.

### 3.9 qRT-PCR validation of RNA-Seq data

**Figure 8:**
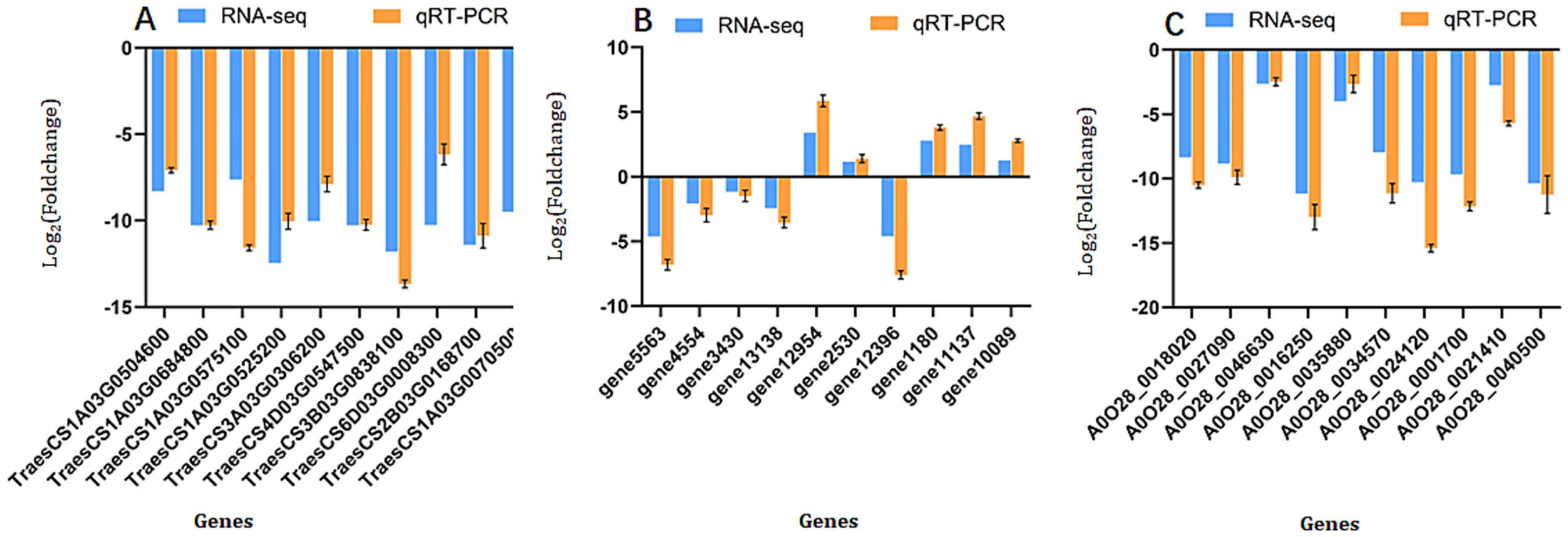
Validation of RNA-seq data by qRT-PCR analysis. Relative expression levels of ten differentially expressed genes (DEGs) in the comparisons of CK vs. Fg **(A)**, Fg vs. Th-Fg **(B)**, and Th vs. Th-Fg **(C)**, respectively. Data are presented as mean ± SD (*n* = 3).

To validate the reliability of our RNA-seq data, we performed qRT-PCR analysis on 30 differentially expressed genes (DEGs)—10 from *T. aestivum*, 10 from *F. graminearum*, and 10 from *T. hamatum*—selected from the most significantly enriched pathways, as detailed in section 3.4–3.7. The selected wheat genes were representative of the key processes identified in the transcriptome: "defense activation” genes (e.g., pathogenesis-related (PR) genes and phenylpropanoid biosynthesis enzymes) and “stress response” genes (e.g., those encoding heat shock proteins (HSPs) and antioxidant enzymes). As shown in Figure 8, the relative expression trends of the selected DEGs measured by qRT-PCR were in strong agreement with the RNA-seq results across all three comparative groups: CK vs. Fg (Figure 8A), Fg vs. Th-Fg (Figure 8B), and Th vs. Th-Fg (Figure 8C). A significant positive correlation (R^2^ > 0.85, *p* < 0.001) was observed between the qRT-PCR and RNA-seq datasets. This high consistency robustly confirms the accuracy and reliability of our transcriptomic analysis, and specifically validates the expression patterns of genes central to wheat defense priming and stress mitigation. This provides a solid foundation for the subsequent mechanistic interpretations.

## 4 Discussion

This study, by integrating *in vitro* antagonism assays with an in planta tripartite interaction transcriptome analysis, systematically elucidates the multi-layered mechanisms through which Th suppresses Fg and mitigates FHB in wheat. Our results support the initial hypothesis and reveal a synergistic model consisting of three core components: direct impairment of pathogen metabolism by Th, priming of the wheat immune system, and alleviation of pathogen-induced physiological damage. The following sections discuss these key findings in depth.

### 4.1 Direct antagonism: *Trichoderma hamatum* suppresses *Fusarium graminearum* by disrupting central metabolic pathways

Our study found that *F. graminearum* exhibited widespread transcriptional suppression when co-inoculated with *T. hamatum* (Th-Fg), with 608 downregulated DEGs significantly enriched in carbohydrate metabolic processes such as polysaccharide catabolic process and xylan catabolic process (Figure 3C). This finding provides strong molecular evidence for the direct antagonistic action of Th, corroborating previous observations that *Trichoderma* species can inhibit *F. graminearum* through direct interactions (Li Z. et al., 2020; Kostrzewska-szlakowska and Kiersztyn, 2017). Carbohydrate metabolism is central to nutrient acquisition, cell wall integrity, and energy generation for fungal pathogens (Zhang et al., 2022). The inhibition of these pathways, including those involving glycosyltransferases (GT), glycoside hydrolases (GH), and polysaccharide lyases (PL) families which are essential for fungal survival and host interaction (Trinca et al., 2023), likely directly contributed to the restricted mycelial growth of Fg observed in the dual-culture assay (Figure 1) and the reduced disease symptoms *in planta* (Figure 2). This aligns with the known efficacy of *Trichoderma asperellum* and *T. hamatum* in inhibiting *Fusarium* growth through mechanisms such as secreting cell wall-degrading enzymes (Li et al., 2020; Ji et al., 2023; Rabuske et al., 2024). However, our transcriptomic data reveal the global nature of this suppression, suggesting that Th′s attack targets a broad spectrum of fundamental metabolic networks in Fg—potentially disrupting glucose uptake, glycolysis, and the pentose phosphate pathway vital for energy production rather than a single pathway. This multifaceted approach extends beyond the action of specific enzymes, such as the cellulase synthesized by *T. koningii* (Pang et al., 2021; Xiang et al., 2021), indicating a more comprehensive antagonistic strategy. The downregulation of carbohydrate metabolism genes could also lead to cascading effects on interconnected cellular processes like amino acid and lipid metabolism, potentially causing a systemic failure of essential metabolic functions in the pathogen (Li et al., 2020). This broad-spectrum disruption of central metabolism likely represents a more efficient and durable antagonistic strategy less prone to resistance development, as it simultaneously undermines the pathogen’s capacity to overcome host defenses, which themselves involve complex carbohydrate metabolism pathways (Tu et al., 2023; Eldakak et al., 2018). The effectiveness of this strategy highlights the potential of *T. hamatum* as a robust biocontrol agent against *F. graminearum*, a major threat to cereal crops (Tu et al., 2023; Liu et al., 2022).

Strikingly, in contrast to the “transcriptional storm” experienced by the pathogen, *T. hamatum* itself displayed minimal transcriptional reprogramming when confronting *F. graminearum* (only 61 DEGs in Th vs. Th-Fg), and these genes were primarily enriched in translation-related functions (Figure 4). This finding suggests a strategic reliance on constitutively expressed antagonistic machinery rather than a massive gene activation response. This minimal transcriptional change implies an evolutionary adaptation where *T. hamatum* may employ a rapid, pre-emptive defense system against *F. graminearum* without incurring the high metabolic cost of extensive *de novo* gene expression and protein synthesis. This observation aligns with the well-documented role of *Trichoderma* spp. as effective biocontrol agents against a wide range of plant pathogens, including *Fusarium* spp. (Feng et al., 2025; Pedrero-Méndez et al., 2025; Karuppiah et al., 2022; Ren et al., 2025; Saravanakumar et al., 2017). Their antagonistic activities, which encompass mycoparasitism, competition, and antibiosis (Yao et al., 2023; Priyashantha et al., 2023), may rely heavily on pre-formed or constitutively produced effectors such as cell wall-degrading enzymes and antibiotics. Specifically, the potent antagonistic effects of *T. hamatum* against various *Fusarium* species, including *F. graminearum* as observed in our and others’ confrontation assays (Feng et al., 2025; Ren et al., 2025; Oviya et al., 2022), can therefore be interpreted as a resource-efficient antagonistic strategy. By maintaining a state of constant readiness, *T. hamatum* can rapidly and effectively suppress pathogen growth, representing a highly efficient biocontrol mechanism.

### 4.2 Plant-mediated defense: *Trichoderma hamatum* primes wheat immune responses for enhanced resistance

The second major finding of this study is that *T. hamatum* alone significantly primes the wheat defense system, inducing a “defense-ready” state characteristic of Induced Systemic Resistance (ISR; Wu et al., 2025; Siddaiah et al., 2017; Dalio et al., 2020). In the absence of the pathogen (CK vs. Th), Th inoculation induced 11,711 wheat DEGs significantly enriched in key defense pathways (Figure 6). The activation of phenylpropanoid biosynthesis, a cornerstone pathway for producing antimicrobial compounds like lignin, phenolics, and flavonoids (Wu et al., 2025; Iriti and Faoro, 2009; Ye et al., 2020), suggests enhanced physical and chemical barrier formation (Yang et al., 2023; Banerjee and Gangopadhyay, 2024). Concurrently, the enrichment of glutathione metabolism indicates an enhanced capacity for scavenging reactive oxygen species (ROS) and maintaining redox homeostasis upon pathogen challenge (Wu et al., 2025; Min et al., 2024; Xu et al., 2019; Wang et al., 2023). Furthermore, the upregulation of alpha-linolenic acid metabolism, a precursor pathway for jasmonic acid (JA) biosynthesis (Min et al., 2024; Jiang et al., 2023), points to a preparedness for JA-mediated defense signaling, which is crucial against necrotrophic pathogens like *F. graminearum* (Wu et al., 2025; Banerjee and Gangopadhyay, 2024; Tian et al., 2024). This multi-faceted priming effect means wheat can mount a faster and stronger defense response upon actual *F. graminearum* invasion.

Our findings corroborate that various *Trichoderma* strains induce plant resistance, but they clearly delineate the specific pathways activated in the FHB pathosystem. It is noteworthy that while *F. graminearum* infection alone (CK vs. Fg) also triggered some defense-related pathways, it occurred alongside a global suppression of gene expression (Figure 5A), implying a potentially dysregulated defense system due to the pathogen’s virulence strategies (Balsells-Llauradó et al., 2020). In contrast, priming by *T. hamatum* prior to stress challenge positions wheat in a more advantageous “alerted” state, enabling a more effective and coordinated defense response. This mechanism, involving intricate crosstalk between defense pathways, underscores the potential of *T. hamatum* as a sustainable strategy to enhance crop resilience against FHB.

### 4.3 Alleviation of pathogen-induced physiological damage: a key mechanism for maintaining plant health

Beyond direct pathogen suppression and defense priming, the third innovative finding of this study is Th′s significant role in mitigating the physiological damage caused by Fg to wheat. The most compelling evidence comes from the comparison between the dual inoculation (Th-Fg) and the control (CK). In stark contrast to the general suppression of genes related to photosynthesis and carbon metabolism observed under Fg infection alone (Figure 5D), these crucial physiological processes, such as “photosynthesis,” “carbon fixation in photosynthetic organisms,” and “carbon metabolism,” were significantly enriched and exhibited enhanced expression in the Th-Fg treatment (Figure 7). This indicates that the presence of Th not only contained pathogen growth but, more importantly, helped wheat maintain or restore its fundamental physiological functions. Our findings reveal that the biocontrol efficacy of *T. hamatum* extends beyond conventional “disease resistance” into the realm of “tolerance” or “health maintenance.” Crucially, *T. hamatum* mitigates the severe physiological damage caused by *F. graminearum* (Fg), particularly its suppression of photosynthesis. Photosynthesis, as the fundamental source of energy and biomass, is critical for plant health and minimizing yield loss (Li et al., 2020). By alleviating the pathogen-induced suppression of photosynthetic and carbon metabolism pathways (Figure 7), *T. hamatum* enables wheat to maintain energy production and resource allocation toward defense and repair processes. This mechanism of enhancing the plant’s intrinsic ability to withstand pathogen pressure—tolerance—differs fundamentally from the mode of action of fungicides that directly kill pathogens (Cheng et al., 2014). The ability of *T. hamatum* to improve plant health likely involves a combination of effects, including reducing pathogen load (Li et al., 2020) and strengthening overall physiological resilience through mechanisms such as enhanced antioxidant systems (Vindas-Reyes et al., 2024; Tan et al., 2024). This approach aligns with the growing importance of sustainable strategies that complement breeding for resistance (Chu et al., 2011; Gyawali et al., 2023; Cai et al., 2005) and move beyond sole reliance on chemical controls (Powell et al., 2024). By fostering plant tolerance through physiological improvement, *T. hamatum* represents a promising component of integrated management strategies against Fusarium head blight.

## 5 Conclusion and future perspectives

In conclusion, this study successfully tested our initial hypothesis that *T. hamatum* suppresses *F. graminearum* and mitigates FHB through a multifaceted mechanism involving both direct antagonism and indirect mediation of wheat defense responses. By employing an integrated tripartite transcriptomic approach, we have provided unprecedented molecular insights into the complex interactions among the biocontrol agent, the pathogen, and the host plant. Our findings confirm that the biocontrol efficacy of *T. hamatum* is achieved through a synergistic triad of mechanisms: it directly “disarms” Fg by extensively suppressing its core metabolic pathways, it “arms” the plant by priming defense pathways such as phenylpropanoid and glutathione metabolism, and it “mitigates damage” by maintaining core physiological functions like photosynthesis, thereby comprehensively enhancing wheat tolerance to FHB. This multi-pronged strategy makes *T. hamatum*a highly promising biocontrol agent against FHB. These mechanistic insights, particularly the identification of key defense pathways (e.g., phenylpropanoid biosynthesis) and stress tolerance genes that are effectively primed or restored by *T. hamatum*, provide concrete molecular targets for innovative breeding programs. Incorporating such genes or modulating these pathways via marker-assisted selection or gene editing could accelerate the development of wheat cultivars with enhanced, pre-formed resistance to FHB, complementing conventional resistance breeding and contributing to more durable and sustainable disease management.

Future research should focus on: (1) Identifying the key constitutively expressed antagonistic effectors in Th; (2) Functionally validating the role of the key pathways proposed herein (e.g., phenylpropanoid metabolism) in Th-mediated resistance using mutants or transgenic plants; (3) Further validating the efficacy of *T. hamatum* and its impact on wheat yield and quality under field conditions. This study provides valuable resources for understanding the molecular dialog in *Trichoderma*-pathogen-plant tripartite interactions and lays a solid theoretical foundation for developing green FHB control strategies based on *T. hamatum*.

## Data Availability

The raw transcriptome sequencing data have been deposited in the national center for biotechnology information (NCBI) database under the BioProject ID PRJNA1356531. Other relevant data supporting the findings of this study are available in this article and its associated Supplementary material.

## Supplementary material

SUPPLEMENTARY TABLE S1 Primer sequences employed in qRT-PCR.

SUPPLEMENTARY TABLE S2 Sequencing data statistics.

SUPPLEMENTARY TABLE S3 Read mapping efficiency to the Fusarium graminearum genome.

SUPPLEMENTARY TABLE S4 Read mapping efficiency to the *Trichoderma hamatum* genome.

SUPPLEMENTARY TABLE S5 Read mapping efficiency to the *Triticum aestivum* genome.

SUPPLEMENTARY TABLE S6 Differentially expressed genes associated with *Fusarium graminearum* in the Fg vs. Th-Fg comparison.

SUPPLEMENTARY TABLE S7 Differentially expressed genes associated with *Trichoderma hamatum* the Th vs. Th-Fg comparison.

SUPPLEMENTARY TABLE S8 Differentially expressed genes associated with *Triticum aestivum* the CK vs. Fg comparison.

SUPPLEMENTARY TABLE S9 Differentially expressed genes associated with *Triticum aestivum* the CK vs. Th comparison.

SUPPLEMENTARY TABLE S10 Differentially expressed genes associated with *Triticum aestivum* the CK vs. Th-Fg comparison.
