# Supplementary material for: Dual RNA-Seq analysis unveils the multifaceted mechanisms of Trichoderma hamatum in the biological control of Fusarium graminearum, the causal agent of wheat fusarium head blight
Source: Front Microbiol. 2026 Jan 16;17:1742203. doi: 10.3389/fmicb.2026.1742203 (PMC12855522; doi:10.3389/fmicb.2026.1742203)
Supplement: SUPPLEMENTARY TABLE S1 — Primer sequences employed in qRT-PCR. [file Table_1.DOCX]

Table S1 Primer sequences employed in qRT-PCR

| gene1180 | XM_011318650.1.F：GAAGAAGCCCATCTCCGTAC |
| --- | --- |
|  | XM_011318650.1.R：AATGCCGCCAAGAACATC |
| gene2530 | XM_011320163.1.F：CCGAACGCCTTCTGACTA |
|  | XM_011320163.1.R：CCTGAACGTGAGCACCAC |
| gene3430 | XM_011323987.1.F：AGTAGTCTATTCGGGTGCTG |
|  | XM_011323987.1.R：TTCTCGCTGTTTGGGTTC |
| gene4554 | XM_011322731.1 .F：CTCCTGCTGTGCTTGGTATG |
|  | XM_011322731.1 .R：CGGAGGGAATGGTAGAGG |
| gene5563 | XM_011325812.1.F：GAAACCTGCACCTACCTCC |
|  | XM_011325812.1.R：TCTAATTCCTCCACTCTGACC |
| gene10089 | XM_011320720.1.F：GGCAGTGTCGTCGTTTCA |
|  | XM_011320720.1.R：CGTCCTCACCCTTGTTGG |
| gene11137 | XM_011326912.1.F：AGGAGTAACAGCATCCGTTCA |
|  | XM_011326912.1.R：CCAGTGTCTGCGTCAGCA |
| gene12396 | XM_011323724.1.F：GATAGCAACGCTTCGGATGA |
|  | XM_011323724.1.R：GCACCACAGGCAAACACG |
| gene12954 | XM_011327970.1.F：TCAAGATTCTACTAGCCCATCC |
|  | XM_011327970.1.R：AGAGTTCAGCCACCTCCC |
| gene13138 | XM_011328827.1.F:ATGAATCAACATTCATATAC |
|  | XM_011328827.1.R:GTATATGAATGTTGATTCAT |
| A0O28_0001700 | XM_024919389.1:100..1161.F：CAGCAGCGAAATCCAGAGC |
|  | XM_024919389.1:100..1161.R：AGCCTCCTGAGCGAAGACC |
| A0O28_0016250 | XM_014085768.2:155..934.F：CATCCGTTACCCTGACCC |
|  | XM_014085768.2:155..934.R：TTACGACCACCAGTGACCAT |
| A0O28_0018020 | XM_073706350.1:5..364.F：GGAATCCTCCAGCAAGGC |
|  | XM_073706350.1:5..364.R：TGAACTCGACACGGACCTG |
| A0O28_0021410 | XM_073698727.1:1..462.F：CCCGAGGCTCTGAAGAAGT |
|  | XM_073698727.1:1..462.R：CTTGGCACCCTTGACCTTT |
| A0O28_0024120 | PKSG01000496.1:<23753..23933.F：AAGTTGGCGCTTGTTATG |
|  | PKSG01000496.1:<23753..23933.R：TTTCTCAGAGGAGGAGTGTT |
| A0O28_0027090 | LVVK01000007.1:2152746..2152782.F：TCGTTTCCACCAAGATGC |
|  | LVVK01000007.1:2152746..2152782.R：AACCTGGTCACCCTCCTC |
| A0O28_0034570 | XM_073700125.1:1..459.F：TCCGTTCTCCACGATGCC |
|  | XM_073700125.1:1..459.R：CGAACTCGCCAATGTAGCC |
| A0O28_0035880 | LVVK01000013.1:1954963..1954968.F：CGTCCGCTATGGTGAGAT |
|  | LVVK01000013.1:1954963..1954968.R：GATCCACTTGAGGCTGATTT |
| A0O28_0040500 | MTYH01000036.1:435558..435710.F：CAAGCCCGTCTTCCACAA |
|  | MTYH01000036.1:435558..435710.R：GAGCAGCACCCTTCGTCT |
| A0O28_0046630 | LVVK01000019.1:63812..64112.F：CGTTGACGGCAAGGACTC |
|  | LVVK01000019.1:63812..64112.R：GGTGGGCACGGATCTTCT |
| TraesCS1A03G0176200 | XM_044602253.1.F：GTGCTTGGATGCTGGTGTCT |
|  | XM_044602253.1.R：GGCTTCAAGAGCAGGTAGACAT |
| TraesCS1A03G0504600 | XM_044472462.1.F：ACGAGTCCTGGAACGTCACCC |
|  | XM_044472462.1.R：CCTTGAGCACCACCGCCAT |
| TraesCS1A03G0525200 | XM_044473362.1.F：ACAGGGATTAGCGTCCACA |
|  | XM_044473362.1.R：CCGCCCAGGTATCAACAG |
| TraesCS1A03G0575100 | XM_044475514.1.F：GCCCGAGGGTGCTCTTTA |
|  | XM_044475514.1.R：GGTGACATTGCCTGCTTCA |
| TraesCS1A03G0578300 | XM_044475777.1.F：CGCCATCGAAATTGCTGAA |
|  | XM_044475777.1.R：CGCCAGGGAAACATACGG |
| TraesCS1A03G0637500 | XM_044478443.1.F：GGCAAGTGGAAAGGAGCA |
|  | XM_044478443.1.R：TCCAAGCCACCGCTGTAA |
| TraesCS1A03G0640500 | XM_044478527.1.F：CCAATTTCGCCGACACTA |
|  | XM_044478527.1.R：TCCTTCCCATCATCACCAT |
| TraesCS1A03G0648100 | XM_044478936.1.F：AGAGGATCAAGAAAGCAGGGAA |
|  | XM_044478936.1.R：AACGGACAACAGTATCGTAGGG |
| TraesCS1A03G0684800 | XM_044480254.1,F：ATCCGAGGTAGAGGGTTT |
|  | XM_044480254.1.R：GCAGCAGCGTAGGAGATA |
| TraesCS1A03G0684800 | XM_044480254.1.F：ATTCGGAACAGCCCACAG |
|  | XM_044480254.1.R：GCTCCATCCAGGTAGTCAAGT |
| β-TUB | F：ACTCCAAGAACATGATGTG |
|  | R：GATCCACTCGACGAAGTA |
| FgEF1A | F：GGCTTTCACCGACTACCCTCCTCT |
|  | R：ACTTCTCGACGGCCTTGATGACAC |
| tef1 | F：TACAAGATCGGTGGTATTGGA |
|  | R：AGCTGCTCGTGGTGCATC |
