# Supplementary material for: Dual RNA-Seq analysis unveils the multifaceted mechanisms of Trichoderma hamatum in the biological control of Fusarium graminearum, the causal agent of wheat fusarium head blight
Source: Front Microbiol. 2026 Jan 16;17:1742203. doi: 10.3389/fmicb.2026.1742203 (PMC12855522; doi:10.3389/fmicb.2026.1742203)
Supplement: SUPPLEMENTARY TABLE S2 — Sequencing data statistics. [file Table_2.DOCX]

Table S2 Sequencing data Statistics

| **Samples** | **Clean reads** | **Clean bases** | **GC Content** | **%≥Q30** |
| --- | --- | --- | --- | --- |
| CK1 | 56,033,050 | 16,764,340,236 | 52.47% | 95.11% |
| CK2 | 52,432,759 | 15,665,724,452 | 53.46% | 95.46% |
| CK3 | 54,096,065 | 16,180,524,789 | 52.78% | 95.29% |
| Fg1 | 52,766,469 | 15,760,457,963 | 54.46% | 95.15% |
| Fg2 | 54,733,498 | 16,343,110,540 | 54.49% | 94.99% |
| Fg3 | 55,966,045 | 16,707,266,505 | 54.52% | 95.14% |
| Th1 | 53,199,088 | 15,879,374,531 | 53.36% | 95.20% |
| Th2 | 51,813,373 | 15,199,301,394 | 53.69% | 95.04% |
| Th3 | 51,878,237 | 15,486,021,301 | 53.52% | 95.28% |
| Th-Fg1 | 55,400,265 | 16,550,265,426 | 54.42% | 94.95% |
| Th-Fg2 | 55,773,641 | 16,564,606,907 | 54.12% | 95.02% |
| Th-Fg3 | 54,761,710 | 16,363,517,501 | 54.22% | 95.08% |

Note:

(1) Samples: Sample name;
(2) Clean reads: Counts of clean PE reads;
(3) Clean bases: total base number of Clean Data;
(4) GC content: Percentage of G, C in clean data.
(5) ≥Q30%: Percentage of bases with Q-score no less than Q30.

(6) CK, mock-inoculated control (wounded but not inoculated); Fg, inoculated with F. graminearum alone; Th, inoculated with T. hamatum alone; Th-Fg, co-inoculated with both F. graminearum and T. hamatum.
